# Energy Harvesting Microelectromechanical System for Condition Monitoring Based on Piezoelectric Transducer Ring

**DOI:** 10.3390/mi16060602

**Published:** 2025-05-22

**Authors:** Kaixuan Wang, Hao Long, Di Song, Hasan Shariar

**Affiliations:** 1 School of Information Engineering, Xuzhou College of Industrial Technology, Xuzhou 221140, China; wang_kaixuan123@163.com; 2 School of Mechatronic Engineering, Jiangsu Normal University, Xuzhou 221116, China; 3 School of Mechanical Engineering, Southeast University, Nanjing 210096, China; 4 Department of Mechanical Engineering, University of Manitoba, Winnipeg, MB R3T 5V6, Canada

**Keywords:** energy harvesting, MEMS, piezoelectric transducer, condition monitoring, shaft bearing

## Abstract

For complex mechanical transmission equipment, shaft bearings are usually enclosed together with the shaft in the internal space of the housing to maintain good sealing and reliability. However, it is difficult to monitor the status of the shaft bearing through external sensors on the housing, while internal sensors face challenges in energy supply and data transmission. Therefore, a piezoelectric transducer ring-based energy harvesting microelectromechanical system (PTR-EH-MEMS) is proposed for the condition monitoring of shaft bearings. Specifically, the piezoelectric transducer ring is designed to convert mechanical vibrations into electrical energy, which simultaneously acts as a self-powered monitoring sensor through energy harvesting. In addition, the MEMS is embedded for piezoelectric data processing and condition monitoring of the shaft bearings. To verify the proposed PTR-EH-MEMS, an experimental investigation is implemented under different conditions. The experimental results demonstrate that the system can achieve the maximum DC output of 0.8 V and the root mean square power of 43.979 μW within 128 s, which can effectively identify early-stage bearing faults frequency through a self-powered mode. By combining energy harvesting with condition monitoring capability, the PTR-EH-MEMS offers a compact and sustainable approach for predictive maintenance in rotating machinery, reducing the reliance on external power sources and enhancing the reliability of industrial systems.

## 1. Introduction

Modern mechanical transmission systems increasingly rely on fully enclosed bearing housing to ensure operational reliability and environmental protection [1]. While this design offers significant advantages in sealing performance, it creates substantial challenges for real-time condition monitoring of critical shaft-bearing components. The effective monitoring of shaft bearing status is of paramount importance since bearings are among the most vulnerable components in rotating machinery, and it directly impacts system reliability and operational safety [2]. The ability to accurately monitor shaft bearing condition in enclosed environments, therefore, represents a key technological challenge with significant economic and safety implications for modern industrial systems.

Based on the characteristics of fault sensitivity and easy installation, the vibration sensor is widely applied to condition monitoring in various fields [3,4,5]. Due to the influence of structural damping and interface losses, traditional external vibration sensors mounted on housing surfaces suffer from severe signal attenuation, and they have poor performance in early-stage defect monitoring for shaft bearings [6]. To overcome the shortcomings of external monitoring, the internal sensor monitoring method is presented by installing sensors inside the device housing, which is widely studied and applied for internal component condition monitoring [7,8,9]. Although this approach is theoretically more accurate, it faces application barriers for sustainable power supply and data transmission under continuous rotation conditions [10]. Enclosing a shaft bearing inside its device further limits its application in shaft bearing monitoring.

To overcome the power limitation challenges in internal bearing monitoring systems, energy harvesting technology has emerged as a promising solution [11]. Specifically, the vibration-based piezoelectric energy harvesting approach has gained particular attention due to its high power density and compatibility with rotating machinery environments [12]. In addition, with the unique advantage of simultaneously harvesting mechanical energy and serving as a sensitive vibration sensor, the piezoelectric transducer is suitable for self-powered condition monitoring applications in closed bearing systems, as shown in several studies [13,14]. However, there are significant challenges in achieving energy conversion efficiency while maintaining sensitive fault detection capabilities. Specifically, the confined space within bearing and equipment housing demands extremely compact designs, and the complex dynamic loading conditions require the careful consideration of transducer placement and orientation [15]. Furthermore, the harvested energy must have the power and capacity requirements for not only the sensing elements but also the necessary signal processing and wireless transmission components [16].

To address these fundamental limitations, this study introduces an innovative piezoelectric transducer ring-based energy harvesting microelectromechanical system (PTR-EH-MEMS) for self-powered condition monitoring. The core innovation of the proposed PTR-EH-MEMS lies in its piezoelectric transducer ring, which can directly convert mechanical vibration into electrical energy while serving as a high-sensitivity vibration sensor. This integrated approach eliminates the need for external power sources, and it maintains a direct measurement capability at the vibration source, which is applied to shaft bearing condition monitoring. Based on this experimental investigation, the performance of the proposed PTR-EH-MEMS is validated through different aspects. The novelty of this research lies in designing the piezoelectric transducer ring for energy harvesting and the proposed self-powered PTR-EH-MEMS for shaft bearing condition monitoring. It is reflected as follows:

1. A ring-shaped piezoelectric transducer is optimized to be embedded in the bearing housing, and the energy from the bearing rolling motion is harvested and stored;
2. A self-powered PTR-EH-MEMS is proposed and installed in the housing of the shaft system, which is applied for shaft bearing condition monitoring based on signal processing and transmission;
3. The experimental investigation is implemented to test and validate the effectiveness of the proposed PTR-EH-MEMS in energy harvesting and condition monitoring.

The remainder of this paper is organized as follows: Section 2 describes the preparation of the proposed system. The experimental investigation is detailed in Section 3, followed by conclusions in Section 4.

## 2. Preparation of Energy Harvesting Microelectromechanical System

### 2.1. Working Principle of Piezoelectric Transducer

**Figure 1:**
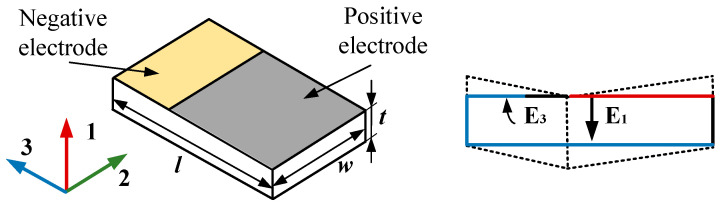
The deformation mode of *d*_31_ for PZT with wrapped electrodes.

The piezoelectric transducer operates based on the direct piezoelectric effect, where mechanical strain induces an electrical charge in some crystalline materials, such as lead zirconate titanate piezoelectric ceramic (PZT) and lead bismuth titanate piezoelectric ceramic (PCT) [17,18]. When subjected to dynamic mechanical stress and vibrations, the deformation of the piezoelectric material causes electric dipole displacements due to its non-centrosymmetric atomic structure, which can generate a measurable voltage across the electrodes [19]. Based on its piezoelectric effect, the piezoelectric transducer is developed with different piezoelectric materials. Specially, the electromechanical coupling behavior is described as follows:

(1) $$\left\{\begin{array}{l} S_{h}=S_{hk}^{E}T_{k}+d_{ih}\cdot E_{j}\;\;\;h,k=1,2,3,4,5,6\\ D_{i}=d_{ik}\cdot T_{k}+\varepsilon_{ij}^{T}E_{j}\;\;\;i,j=1,2,3 \end{array}\right.,$$

where *D_i_* is the electric displacement, *d_ik_* is the piezoelectric strain coefficient, *T_k_* is the mechanical stress, *S_h_* is the mechanical strain, *E_j_* is the electric field, *ε_ij_^T^* is the permittivity under constant stress, and *S_hk_^E^* is the elastic compliance under constant electric field. Specially, the indices *i* and *j* refer to electrical quantities in different directions, and *h* and *k* are the mechanical quantities in different directions. For the roller movement process of the shaft bearing, taking into account centrifugal force and gravity, the *d*_31_ direction is the most significant mode of change for the piezoelectric transducer. It is also the main method of power generation, as shown in Figure 1.

For energy harvesting applications, the voltage output *V* of a piezoelectric patch under dynamic bending can be expressed as follows:

(2) $$V=\frac{d_{31}Y_{P}t_{P}A\ddot{x}}{C_{p}},$$

where *V* is the voltage output, *d*_31_ is the transverse piezoelectric coefficient, *Y_p_* is the Young’s modulus of the piezoelectric material, *t_p_* is the thickness of the piezoelectric layer, *A* is the surface area subjected to strain, $\ddot{x}$ is the acceleration of vibration input, and *C_p_* is the capacitance of the piezoelectric element.

### 2.2. Piezoelectric Transducer Ring Design

**Figure 2:**
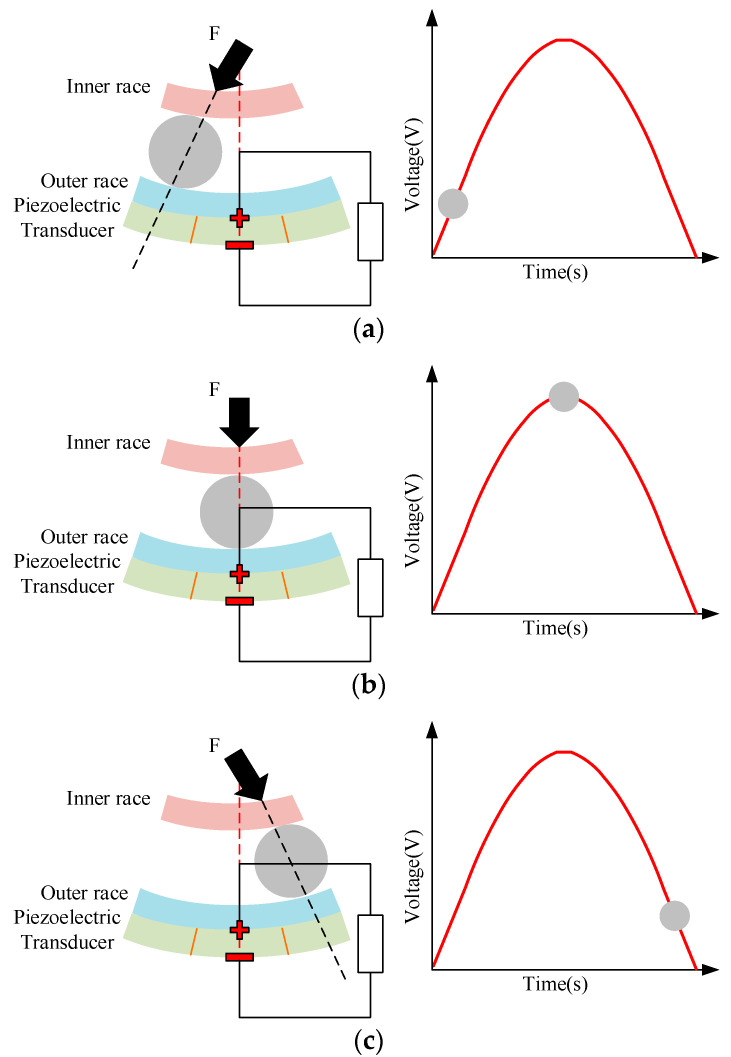
Three distinct phases of energy harvesting under the ball rolling moment: (**a**) approach phase; (**b**) maximum loading phase; (**c**) release phase.

For the ball rolling motion, energy harvesting is implemented through the interaction between the rolling ball bearing and the cut section. As each rolling ball bearing traverses the cut section, the consistent load obtained from the shaft induces dynamic deformation, generating electrical energy through the direct piezoelectric effect. It includes three distinct phases, approach phase, maximum loading phase, and release phase, as shown in Figure 2 and as follows:

1. Approach phase: The rolling ball enters the cut section and the initial compressive stress is created at the leading edge of the piezoelectric element. This leads to an increase in the voltage output, as illustrated in Figure 2a;
2. Maximum loading phase: As the rolling ball progresses and reaches the midpoint of the cut section, the voltage attains its peak level and the maximum load is exerted on the section, as depicted in Figure 2b. Specifically, the power output is maximized at the maximum loading phase and is expressed as follows:

(3) $$P_{\max}=\frac{1}{2}k^{2}\omega m{\ddot{x}}^{2},$$

where *P*_max_ is the maximum power output, *k* is the electromechanical coupling factor, *ω* is the angular frequency of vibration, and *m* is the effective mass of the vibrating structure;

3. Release phase: As the rolling ball exits the cut section, the piezoelectric element is restored from the compressed condition and the voltage output begins to decrease, as shown in Figure 2c.

**Figure 3:**
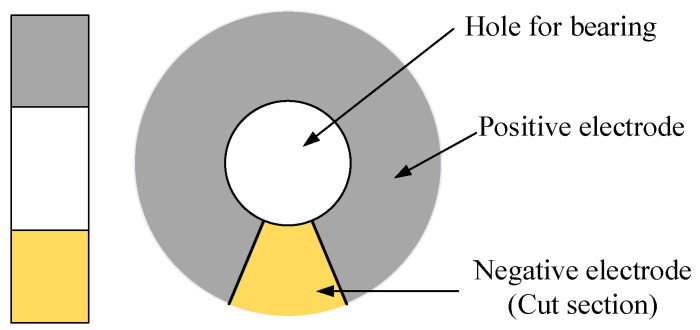
The structural of the piezoelectric transducer ring.

The piezoelectric transducer ring, which has a hole of the same size as the outer ring of the bearing, is designed based on the characteristics of piezoelectric transducers and the structure of shaft bearings. The thickness of the piezoelectric transducer ring is slightly larger than that of the bearing, as shown in Figure 3. Specifically, the ball bearing passes through the angular cutting portion on the piezoelectric transducer ring and generates fatigue load, which is transmitted from the ball bearing and the housing to the tightly fitted piezoelectric transducer ring. Then, the loaded ball transfers the load to the bearing seat, creating a cyclic loading and unloading mode to achieve maximum AC output. The applied forces include gravity, centrifugal force, and a slightly unbalanced force. These combined forces contribute to generating output energy and enhancing the performance of energy harvesting.

### 2.3. Piezoelectric Transducer Ring-Based Energy Harvesting Microelectromechanical System

**Figure 4:**
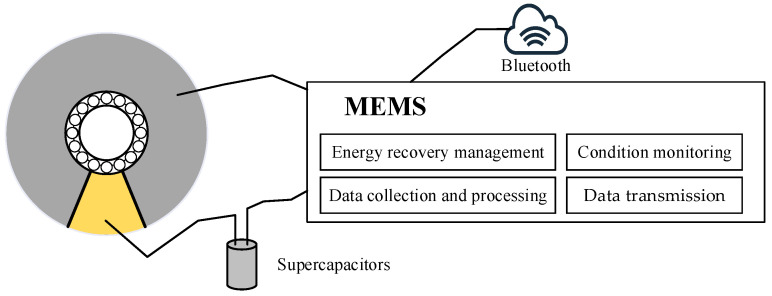
The workflow and flowchart of the proposed PTR-EH-MEMS.

To efficiently harvest and utilize the generated energy, a PTR-EH-MEMS is designed for self-powered condition monitoring. The system includes several significant parts: supercapacitors, energy recovery management model, data collection and processing model, data transmission model, and so on. The supercapacitor used in the proposed PTR-EH-MEMS is employed for its balanced performance in energy density, power density, and compact form factor, which aligns with the spatial constraints of bearing housing. It can store the harvested energy for a stable power supply and it is used to process and transmit data through MEMS at specific times. In addition, the energy recovery management model consists of a customized power management circuit (PMC) to optimize energy transfer between the piezoelectric transducer ring and the supercapacitor. The PMC has three key subsystems, including the active rectification stage, maximum power point tracking, and voltage regulation module. The PMC is employed for active rectification with high efficiency and it can achieve maximum power point tracking to optimize energy extraction. For the data collection and processing model, the low-power MEMS microcontroller is applied for signal conditioning and real-time Fast Fourier Transform analysis to detect bearing fault frequency. In addition, Bluetooth low energy technology is used for intermittent data upload in the data transmission model. The workflow of the proposed PTR-EH-MEMS is shown in Figure 4.

When a shaft bearing has a fault, it generates a periodic impulse in the signal. Since bearings are standard components, the condition of a bearing is monitored by identifying the bearing fault frequency, including three fault types: inner ring fault, outer ring fault, and rolling element fault [20,21], which are expressed as follows:

(4) $$BPFO=\frac{Z_{n}}{2}f_{r}\left(1-\frac{d\cos\alpha }{D}\right),$$

(5) $$BPFI=\frac{Z_{n}}{2}f_{r}\left(1+\frac{d\cos\alpha }{D}\right),$$

(6) $$BSF=\frac{1}{2}f_{r}\frac{D}{d}\left[1-{\left(\frac{d\cos\alpha }{D}\right)}^{2}\right],$$

(7) $$SF=f_{r}=\frac{N}{60},$$

where *BPFO*, *BPFI*, *BSF*, and *SF* are the inner ring fault, outer ring fault, rolling element fault, and shaft rotating frequency, respectively, *Z_n_* is the number of balls, *f_r_* is the shaft frequency, *d* and *D* are the ball diameter and pitch diameter, respectively, *α* is the contact angle, and *N* is the rotational speed.

Specifically, the amplitudes of *BPFO*, *BPFI*, and *BSF* are identified from a signal frequency spectrum. To avoid theoretical and practical deviations, the maximum frequency within the fault frequency range of ±30 Hz is taken as the fault frequencies of *BPFO*, *BPFI*, and *BSF*. It is defined as three potential fault frequencies. In addition, the standard fault frequencies are obtained based on bearings with three types with artificially induced standard defects, which are expressed as follows:

(8) $$\begin{array}{l} A_{pi}=\max\{\left[F_{pi}-30,F_{pi}+30\right]\}\\ A_{si}=\max\{\left[F_{si}-30,F_{si}+30\right]\}\\ i=BPFO,BPFI,BSF \end{array},$$

where *A_pi_* and *A_si_* are the amplitude of potential and standard fault frequencies, respectively, and *F_pi_* and *F_si_* are the potential and standard fault frequencies, respectively.

If the potential fault frequency amplitudes of *BPFO*, *BPFI*, and *BSF* are higher than the standard fault frequencies, the fault is diagnosed and the fault type is further determined by analyzing the frequency range of the fault, which is expressed as follows:

(9) $$\left\{\begin{array}{l} Fault:A_{pi}\geq A_{si}\\ Normal:A_{pi}<A_{si} \end{array}\right.,$$

Based on the PTR-EH-MEMS design, the piezoelectric transducer ring simultaneously harvests energy and collects vibration signals for self-powered condition monitoring of a shaft bearing. The flowchart of the proposed PTR-EH-MEMS includes energy harvesting, data collection and processing, and self-powered condition monitoring, which are explained in more detail as follows:

1. Energy harvesting: As each rolling ball passes through the cut section, the alternating current is generated based on dynamic deformation of piezoelectric transducer ring. This process follows the conditions of the energy recovery management model for energy harvesting;
2. Data collection and processing: The low-power MEMS microcontroller is employed to acquire and process vibration data from the designed piezoelectric transducer ring at certain intervals. The embedded signal processing unit performs real-time Fast Fourier Transform to extract characteristic frequency components;
3. Self-powered condition monitoring: By analyzing the bearing faults frequency, the condition monitoring is applied to the shaft bearing. When sufficient energy is stored in the capacitors, Bluetooth is activated to upload processed condition results to nearby gateways, and the device can achieve complete energy autonomy through the harvested energy of the proposed PTR-EH-MEMS.

## 3. Experimental Investigation

### 3.1. Experimental Setup

**Figure 5:**
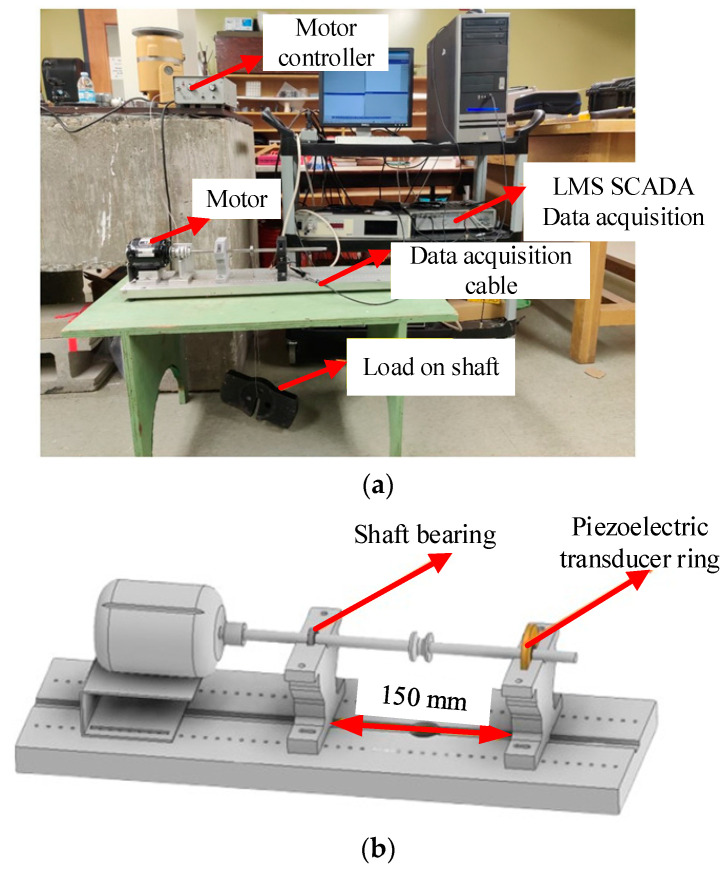
The shaft bearing experimental platform: (**a**) physical image; (**b**) 3D simulation model.

To test the effectiveness of the proposed PTR-EH-MEMS in energy harvesting and condition monitoring, a shaft bearing experimental platform is established for experimental implementation, as shown in Figure 5.

The experimental setup consists of a dual-pedestal test rig designed for comprehensive performance evaluation. The motor-side pedestal houses a standard support bearing, while the opposite pedestal integrates the proposed PTR-EH-MEMS. A balanced loading mechanism applies controlled radial forces to both pedestals simultaneously, ensuring uniform stress distribution across the test bearing. In addition, the rotational speed is regulated by the motor drive controller and the LMS data acquisition module is employed to collect data related to energy harvesting.

To test the designed piezoelectric transducer ring, the shaft bearing seat is assembled with shaft bearing, bushing, piezoelectric transducer ring, and so on. Specifically, the 6656k7 ultra-thin ball bearing is embedded in the piezoelectric transducer ring with a 0.5 mm gap and the parameters of the bearing are shown in Table 1.

**Figure 6:**
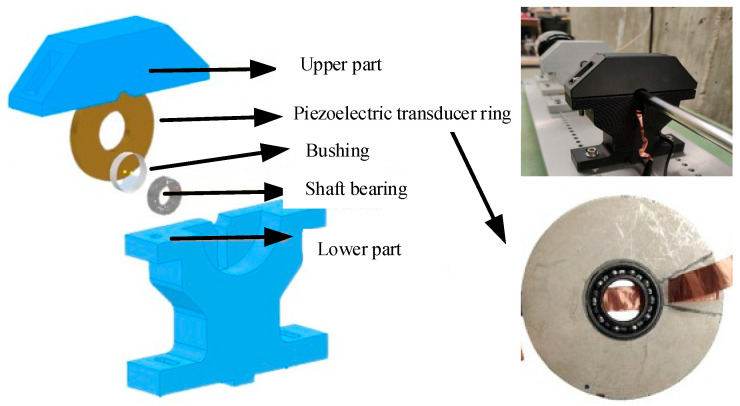
The designed shaft bearing seat.

Notably, a 3D-printed plastic bushing is interposed between the shaft bearing and the piezoelectric transducer ring for secure connection. Additionally, a stainless-steel shaft passes through the shaft bearing and is driven by a motor. The designed shaft bearing seat is shown in Figure 6.

The supercapacitors employed in this experimental setup are the Xuansn XE1 based on a solid-state capacitor. The parameters of the supercapacitors are shown in Table 2.

### 3.2. Experimental Implementation Process

The experiment is conducted under the centered load of 40 lb (17.8 kg) with uniform distribution. The shaft is systematically rotated with speeds of 1000, 2000, 3000, and 4000 rpm, spanning the typical operational range of industrial bearings. By measuring the charging characteristics of three capacitor values (10 μF, 56 μF, and 270 μF), the practical energy storage capabilities are evaluated with three quantified key performance factors, including time-to-voltage thresholds, maximum attainable voltage, and charge/discharge cycle efficiency.

In addition, the experimental setup maintained rigorous environmental controls, with temperature stabilized at 23 ± 1 °C and all test conditions held within ±2% tolerance for load and speed parameters to ensure measurement consistency. Each test condition captured at least 30 complete rotation cycles to guarantee statistically significant results.

### 3.3. Effect of Energy Harvesting

**Figure 7:**
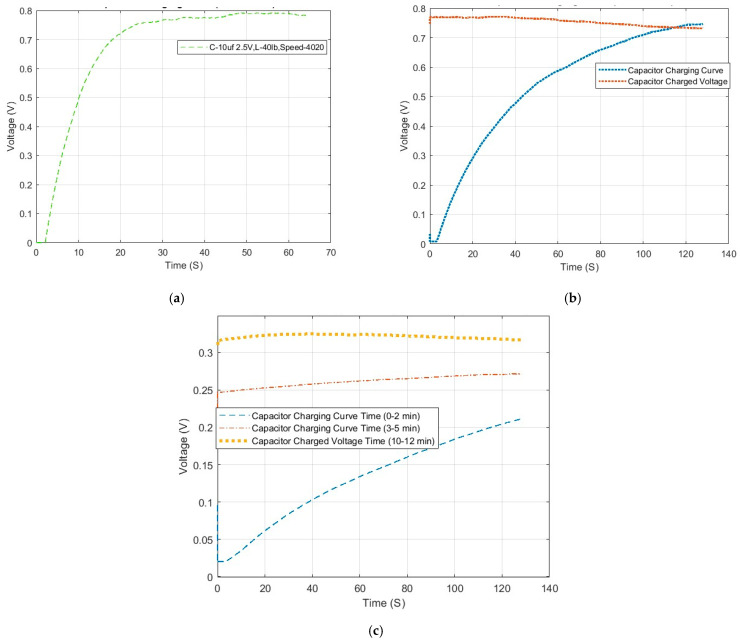
Charging curve and charged voltage curve at 4000 rpm: (**a**) 10 μF; (**b**) 56 μF; (**c**) 270 μF.

For the three capacitors of 10 μF, 56 μF, and 270 μF, the shaft is rotated at 4000 rpm to test the effect of energy harvesting. The charging time is controlled to reach the maximum value for three types of capacitors and the results are shown in Figure 7.

For the 10 µF capacitor, it takes roughly 30 s to reach its maximum voltage of 0.8 V. Furthermore, the root mean square (RMS) power is calculated and it can reach 24.25 μW for 30 s [22]. It is expressed as follows:

(10) $$P_{RMS}=\sqrt{\frac{\sum_{i=1}^{n}V_{i}^{2}}{n}},$$

where *P_RMS_* is the RMS power, *V_i_* is the *i*-th voltage, and *n* is the number of voltage data.

For the 56 µF capacitor, it requires approximately 128 s to reach its maximum voltage of approximately 0.75 V. The second flat line indicates that the capacitor is fully charged and maintains the same charged state. Specifically, it takes approximately 150 s from the initiation of capacitor charging to commence recording data for the fully charged voltage. The RMS power for this charging curve is 43.979 μW in the 128 s.

Due to its large capacitance, the 270 µF capacitor takes approximately 10 to 12 min to reach its maximum charge. The RMS power is 20.60 μW for this charging curve in the 128 s, which is lower than the 56 µF capacitor over the same time span. Importantly, the shaft is observed to rotate continuously, which ensures sufficient time for energy harvesting through the proposed PTR-EH-MEMS.

**Figure 8:**
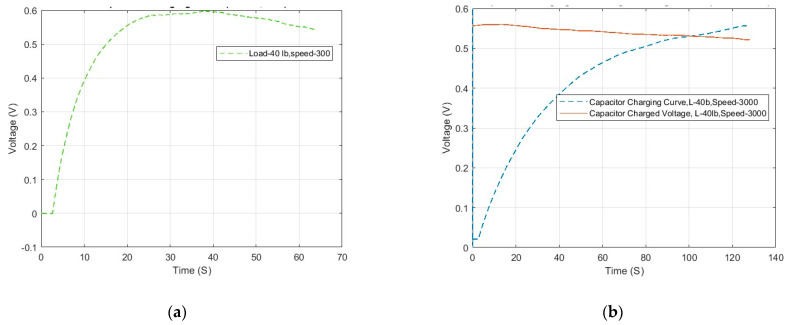
Charging curve and charged voltage curve at 3000 rpm: (**a**) 10 μF; (**b**) 56 μF.

Subsequently, the rotational speed of the shaft is changed to 3000 to analyze the impact of rotational speed on energy harvesting—the results for the 10 μF and 56 μF capacitors are shown in Figure 8. It is notable that the graphs exhibit similarities with the result for a rotational speed of 4000 rpm. For the 10 µF capacitor, the curve starts to trend downward after reaching the maximum DC voltage output of 0.6 V. This is attributed to loosening connections or voltage leakage in the system. Regardless, the calculated RMS power is 14.26 μW in the 30 s charging time. For the 56 µF capacitor, the charging curve displays expected results, requiring approximately 120 s to fully charge the capacitor. Similarly, the charged voltage flatline displays a downward trend, possibly due to voltage leakage. The calculated RMS power for the 56 µF capacitor is 23.82 μW during this charging period.

Overall, the low rotational speed caused the maximum voltage to drop to 0.6 V. The reason for this is that the small rotational speed reduces the centrifugal force and further reduces the load applied by the shaft bearing on the piezoelectric transducer ring, thereby affecting the energy harvesting effect. In practical use, the speed and load can be increased to amplify the load on the piezoelectric transducer ring for high charging efficiency.

In addition, the energy harvesting efficiency (*η*) of the proposed system is quantified through experimental measurement and is expressed as follows:

(11) $$\eta =\left(\frac{P_{E}}{P_{M}}\right)\times 100\%,$$

where *P_E_* is the harvested RMS power that is measured through experiment, and *P_M_* is the mechanical power input, which is expressed as follows:

(12) $$P_{M}=F\cdot v,$$

where *F* is the radial load, and *v* is the vibration velocity derived from measured displacement.

Based on the experimental results, the energy harvesting efficiency is calculated for different conditions, including four rotating speeds and three capacitor values with the same load of 40 lb (17.8 kg) and a temperature of 23 ± 1 °C. To ensure that the output values are easy to observe and analyze, the fixed test conditions are 4000 rpm and 270 μF for rotational speed and capacitor value, respectively. The results are shown in Table 3.

The experimental results reveal critical insights into the energy harvesting performance under varying conditions. For capacitor configurations, the 10 µF capacitor achieved the highest average efficiency of 7.43%, benefiting from its rapid charging dynamics that minimize energy loss during intermittent vibration inputs. Due to prolonged charging times and increased leakage currents inherent to larger capacitances, the capacitors of 56 µF and 270 µF exhibited reduced efficiencies of 6.87% and 5.96%, respectively.

In addition, the rotational speed significantly influenced system performance, with efficiency improving from 4.21% at 1000 rpm to 6.76% at 4000 rpm. This trend correlates with enhanced mechanical energy input at higher speeds, where increased centrifugal forces amplify stress on the piezoelectric transducer ring. For industrial scenarios, the capacitor selection can be optimized based on target rotational speeds and energy storage requirements.

To discuss the impact of speed on energy harvesting, the relationship between speed changes and mechanical excitation frequency is calculated. The mechanical excitation frequency is directly proportional to the rotational speed and the number of rolling elements, which is expressed as follows:

(13) $$f_{ex}=\frac{N}{60}Z_{n},$$

where *f_ex_* is the mechanical excitation frequency.

For the 6656k7 ultra-thin ball bearing with 10 balls, the mechanical excitation frequency is 166.7 Hz, 333.3 Hz, 500 Hz, and 666.7 Hz for rotation speeds of 1000 rpm, 2000 rpm, 3000 rpm, and 4000 rpm, respectively. Specifically, the natural frequency of the proposed system is approximately 600 Hz according to finite element analysis. This implies that an excitation frequency of 4000 rpm approaches the resonance, amplifying strain energy in the piezoelectric transducer. It is also the reason why the rotation speed of 4000 rpm has the highest energy harvesting efficiency. By adjusting bearing geometry, the resonance can shift to align with target rotation speeds and operating the proposed system near resonance can maximize energy harvesting in speed-stable applications.

Due to its inherent electrical characteristics and dynamic interaction with the energy harvesting process, capacitor selection directly impacts energy collection efficiency. Specifically, energy collection efficiency is governed by the relationship between the output impedance of the piezoelectric transducer and the capacitor, which is expressed as follows:

(14) $$\eta =k_{z}\frac{Z_{cap}}{Z_{pie}+Z_{cap}},$$

where *k_z_* is the impedance factor, and *Z_cap_* and *Z_pie_* are the impedance of the piezoelectric transducer and the capacitor, respectively.

The small 10 µF capacitor exhibits high impedance at low frequencies, resulting in power transfer inefficiency with a high 7.43% instantaneous efficiency, yet the rapid charging (τ = RC) minimizes energy loss during transient vibrations. Additionally, the medium 56 µF capacitor achieves optimal impedance matching in the operational frequency range and balancing charge retention and leakage to deliver a peak sustained efficiency of 6.87%. Conversely, the large 270 µF capacitor suffers from severe impedance mismatch and prolonged charging, where extended charging durations amplify leakage losses, reducing efficiency to 5.96%. Thus, mid-range capacitors best harmonize electrical and mechanical time constants for practical energy harvesting.

In addition, the equivalent series resistance and leakage current critically influence efficiency, which can be expressed as follows:

(15) $$P_{loss}=I_{leak}^{2}R_{ESR}+\frac{V_{c}^{2}}{R_{leak}}$$

where *I_leak_* is the leakage current, *R_ESR_* is the equivalent series resistance, *R_leak_* is the leakage resistance of the capacitor, and *V_c_* is the voltage at both ends of the capacitor.

The electrical characteristics of the capacitors significantly impact energy harvesting performance. The 10 µF capacitor, with its low equivalent series resistance, minimizes resistive losses during rapid charge/discharge cycles. However, its high leakage current limits sustained energy retention. The 56 µF capacitor strikes an optimal balance, combining moderate equivalent series resistance and leakage resistance, which ensures efficient energy storage with minimal dissipation over time. In contrast, the 270 µF capacitor exhibits poor performance due to high equivalent series resistance and severe leakage, where resistive losses dominate and significantly reduce overall efficiency. These tradeoffs highlight the critical role of capacitor selection in balancing transient response and long-term energy retention for self-powered systems.

### 3.4. Effect of Condition Monitoring

Based on the rotational speed and bearing parameters, the fault frequency of shaft bearings is calculated for condition monitoring, including BPFO, BPFI, BSF, and SF. The theoretical results are calculated for four rotational speeds through Equations (4)–(7). The results are shown in Table 4.

**Figure 9:**
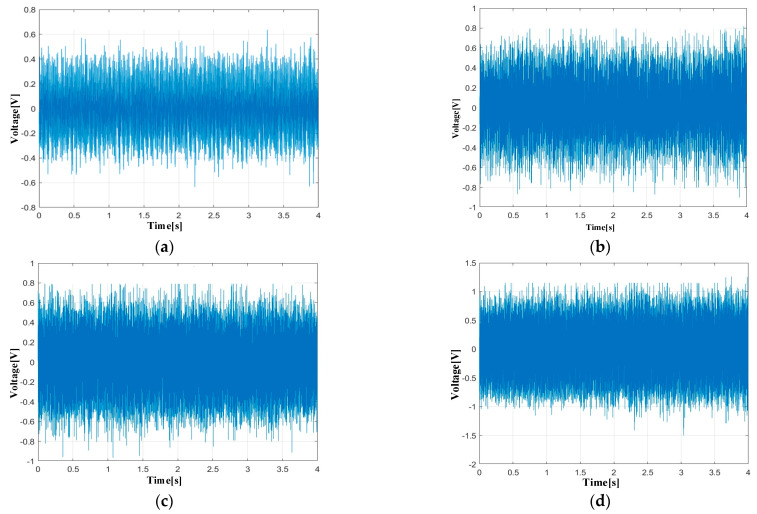
Monitoring results in time domain: (**a**) 1000 rpm; (**b**) 2000 rpm; (**c**) 3000 rpm; (**d**) 4000 rpm.

**Figure 10:**
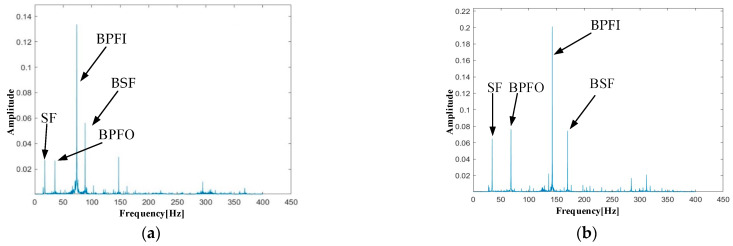

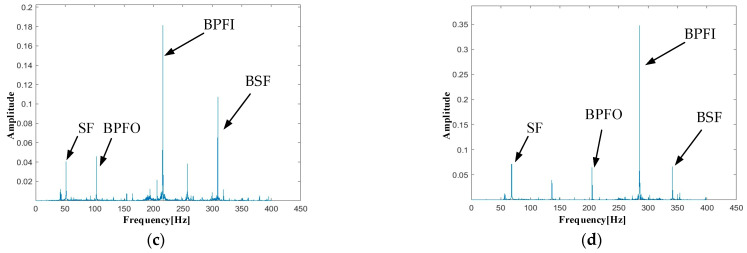
Monitoring results in frequency domain: (**a**) 1000 rpm; (**b**) 2000 rpm; (**c**) 3000 rpm; (**d**) 4000 rpm.

Based on the proposed PTR-EH-MEMS, the condition of shaft bearings is monitored for different rotational speeds. The results in time domain and frequency domain are shown in Figure 9 and Figure 10.

The time domain signals captured by the proposed system clearly demonstrate increasing amplitude with rotational speed, while maintaining consistent waveform characteristics that indicate stable bearing operation. Particularly at 4000 rpm, the periodic impacts corresponding to rolling element passage become more pronounced, yet the overall signal pattern remains regular.

In addition, the frequency domain analysis illustrates the capability of the proposed system in revealing fault frequency, which is significant for the condition monitoring of shaft bearings. As the rotational speed increases, the fault frequency of BPFO, BPFI, BSF, and SF gradually increases. However, overall, the spectrum is clean without noise interference, and the four fault frequencies are clearly visible in the frequency domain. The signal-to-noise ratio remains excellent even at the highest speed, without spectral smearing and aliasing. This is crucial for the early detection of bearing defects and condition monitoring.

The monitoring results validate the dual functionality of the proposed PTR-EH-MEMS in both energy harvesting and precise condition monitoring. The frequency spectra consistently show energy concentration at the theoretically predicted fault frequencies, confirming the accuracy of bearing health assessment. This performance demonstrates that the proposed PTR-EH-MEMS has the potential for reliable, self-powered condition monitoring in practical industrial applications and is capable of detecting incipient bearing faults while operating entirely on harvested energy.

### 3.5. Comparison with Traditional Solutions

Compared with the traditional solutions for energy harvesting and condition monitoring, the proposed PTR-EH-MEMS addresses two critical limitations of prior work, including the spatial constraints of bearing housing and the multifunctionality of energy harvesting and condition monitoring. For traditional monitoring systems, the external vibration sensors and battery-powered internal sensors are usually employed for their convenience. The compared results are shown in Table 5.

The PTR-EH-MEMS eliminates signal attenuation issues inherent to external sensors while avoiding the maintenance demands of battery-powered internal systems. The proposed system integrates the advantages of two traditional solutions to collect high-quality monitoring signals in a continuous and stable way. In addition, the proposed system is compared with traditional energy harvesting solutions. Due to the use of similar piezoelectric materials, the proposed system ensures the same function and efficiency of energy harvesting, which has the additional function of condition monitoring. Additionally, the designed piezoelectric transducer ring is seamlessly integrated into standard bearing housing without mechanical interference.

Due to difficulties in simulating early faults, conventional bearing faults are simulated to establish a baseline performance of the proposed PTR-EH-MEMS, including inner ring faults, outer ring faults, and rolling element faults. While these controlled defects do not fully replicate natural early-stage degradation mechanisms, they provide a systematic framework for system functionality evaluation. Although actual micro-defects are not physically implemented, the spectral features of actual bearing defects (such as local pits, surface cracks, etc.) can be estimated through dynamic system models. For localized pits, the sideband modulation around characteristic frequencies can be introduced with an amplitude proportional to defect size. On the condition monitoring spectrum, there are a series of sidebands around the fault frequency. This illustrates the potential early fault identification for the proposed system.

## 4. Conclusions

This paper proposes a PTR-EH-MEMS for energy harvesting based on a designed piezoelectric transducer ring. Self-powered condition monitoring is also realized for shaft bearings. The performance of the proposed PTR-EH-MEMS is validated through a shaft bearing experimental platform. Experimental results show that the PTR-EH-MEMS can achieve a maximum DC output of 0.8 V and an RMS power of 43.979 μW within 128 s, which can simultaneously provide accurate bearing health monitoring. In addition, the energy harvesting performance is speed-dependent, with higher rotational speeds yielding better power outputs, while the condition monitoring capability remains robust across the entire operational range. Based on the proposed PTR-EH-MEMS, the autonomous monitoring system is realized with high accuracy and without external power sources.

Future work will focus on optimizing systems to achieve higher voltage output and energy harvesting. Furthermore, accelerated fatigue tests will be developed to generate natural subsurface cracks and advanced fault diagnosis algorithms will be implemented to enhance the sensitivity of early fault signature detection and improve its practical applicability in industrial environments.

## Figures and Tables

**Table 1:** The parameters of the shaft bearing.

Parameters	Value
Outer diameter	49.4 mm
Inner diameter	19.5 mm
Thickness	5 mm
Number of balls	10
Diameter of rolling ball	2.4 mm
Pitch diameter	14.5 mm

**Table 2:** The parameters of the supercapacitors.

Parameters	Value
Model	Xuansn XE1
Rated voltage	2.5 V
Temperature range	−55 °C to +105 °C
Feature	Solid-state capacitor

**Table 3:** The results of energy harvesting efficiency.

Different Conditions	Value	Fixed Test Condition	Average Efficiency
Three capacitor values	10 μF	4000 rpm	7.43%
	56 μF		6.87%
	270 μF		5.96%
Four rotating speeds	1000 rpm	270 μF	4.21%
	2000 rpm		5.13%
	3000 rpm		5.97%
	4000 rpm		6.76%

**Table 4:** The theoretical results for information frequency.

Rotational Speed	Bearing Fault Frequency	Value
1000 rpm	BPFO	48.8 Hz
	BPFI	69.3 Hz
	BSF	96.8 Hz
	SF	16.7 Hz
2000 rpm	BPFO	97.9 Hz
	BPFI	139.1 Hz
	BSF	194.2 Hz
	SF	33.3 Hz
3000 rpm	BPFO	147.2 Hz
	BPFI	208.6 Hz
	BSF	291.7 Hz
	SF	50 Hz
4000 rpm	BPFO	196.0 Hz
	BPFI	278.2 Hz
	BSF	388.7 Hz
	SF	66.7 Hz

**Table 5:** Comparison with traditional monitoring systems.

Feature	Proposed System	External Vibration Sensor	Battery-Powered Internal Sensor
Power Source	Self-powered	External power	Battery-powered
Installation	Internal mounting	External mounting	Internal mounting
Signal fidelity	Direct vibration source (SNR > 40 dB)	Attenuated signals (SNR < 20 dB)	Moderate SNR (25–30 dB)
Operational lifetime	Theoretically infinite	Theoretically infinite	Battery life

## Data Availability

Data are available on request.
